# Spatiotemporal tensor analysis for effective information mining of hydraulic structures considering environmental excitation and vibration response

**DOI:** 10.1038/s41598-025-99422-w

**Published:** 2025-05-02

**Authors:** Hui Li, Zhang Han, Tengfei Bao, Xiaohan Duan, Guang Yang, Xianyu Xiong, Yibo Ouyang, Jiankang Lou

**Affiliations:** 1 Department of Hydraulic Engineering, Henan Vocational College of Water Conservancy and Environment, Zhengzhou, 450008 Henan China; 2 College of Architecture and Civil Engineering, Kunming University, Kunming, 650214 Yunnan China; 3 College of Water Conservancy and Hydropower Engineering, Hohai University, NanJing, 210098 Jiangsu China; 4 School of Water Conservancy, North China University of Water Resources and Electric Power, Zhengzhou, 450046 Henan China; 5 School of Engineering Management and Real Estate, Henan University of Economics and Law, Zhengzhou, 450000 Henan China; 6 Henan Water Planning and Design Research CO., LTD., Zhengzhou, 450002 Henan China

**Keywords:** Hydraulic engineering structures, Concrete structures, Damage diagnosis, Nonlinear dynamic systems, Spatiotemporal tensors analysis, Effective information, Civil engineering, Engineering

## Abstract

The vibration response data is a key foundation of vibration-based hydraulic structures’ online damage diagnosis. However, the measured data is often subject to various noises and invalid information, which reduces the accuracy of damage diagnosis, leading to misjudgment and omission of structure damage. The hydraulic structure system is an open, dissipative, and complex nonlinear dynamic system, where at least one or more, or even all parts, have nonlinear interactions. The service condition of hydraulic concrete structures is influenced by environmental factors such as temperature, water temperature and water level. The feature of “open” is mainly manifested as the coupling effect field of multiphase environmental factors. The single-point signal denoising based effective information mining method can lead to over-denoising or under-denoising issues, resulting in low effective information mining accuracy. To overcome these limitations, this paper studies the synchronous denoising technology of multi-point vibration response data, and an improved adaptive variational mode decomposition method was introduced to convert the multi-point vibration response data into a three-dimensional multi-scale spatiotemporal tensor. The factor filter sets construction method based on the Crank–Nicolson-like criterion for fast orthogonal Tucker factor updating method was proposed to denoise the signal preliminary. The time-weighted modified dynamic time warping theory and curvature smoothing algorithm were combined to construct the optimal filter model with a balancing factor to extract the effective information from vibration response. Finally, the data from the sluice model experiment was used to demonstrate the validity of the method proposed in this paper. This article is about the theme of health monitoring for hydraulic concrete structures in the field of civil engineering.

## Introduction

To develop and utilize water resources rationally and efficiently, the scale of various dams, sluices, and embankments has made unprecedented breakthroughs. The hydraulic structure service for decades of life, is usually accompanied by complex load conditions, material itself, and a series of problems, hence structure damage is inevitable. The damage results in great threat to engineering safety, it is not only directly related to its economic benefits, but also directly affect the downstream people life and property safety. Hence the problem of engineering safety is related to the national economy and people’s livelihood event^1^. The working conditions of hydraulic concrete structures are complex and changeable. In its whole life cycle, it is not only subjected to the combination of reservoir water pressure, temperature change and sediment pressure, but also affected by physical and chemical reactions such as freeze–thaw, erosion and alkali aggregate reaction caused by environmental factors. Under the synergistic action of the above factors, the micro-scale defects of the structure will be caused, and the defects will gradually evolve into macro-cracks that endanger the integrity of the structure, and the service behavior of the structure will be deteriorated. A large number of engineering practices have shown that both the existing and under construction concrete structures are affected by cracks. Therefore, the expansion of cracks is the key precursor of structural damage and increases the potential risk of engineering accidents. In many hydraulic concrete structural projects at home and abroad, the cases of engineering failure, abnormal working state and reduced engineering benefit caused by cracks are very common. Due to the left bank shoulder rock fracture development of the Xianghongdian gravity arch dam, serious cracks appear in the dam body. Vertical cracks and radial cracks appeared in more than 20 dam sections upstream of the dam, which affected the normal operation of the project^2^. During the first regular safety inspection of the Baishan gravity arch dam, 141 obvious cracks were found in 7 corridors of 6 floors of the dam, and most of the cracks were in water seepage. The existence of water seepage poses a great threat to the safety of the project^3^. In the first quality inspection of the construction period of the project of Danjiangkou, as many as 2463 cracks were found, among which 17 penetrating cracks with great harm, resulting in long-term water seepage of the dam. These cracks have become a potential safety hazard for dam operation^4^. The above engineering accidents reveal that it is important to diagnose the damage to the hydraulic structure online and understand the service status of the structure in real time.

Traditional damage diagnosis methods are mostly suitable for the diagnosis of local structural damage, so it is difficult to diagnose the overall structural damage. The vibration parameters can simultaneously reflect the local and overall damage state of the structure, so the diagnostic method of structural damage based on the vibration response information has been widely used in recent years. To realize hydraulic structure based on vibration response information of online damage diagnosis, vibration response data is the foundation, but the measured data is usually disturbed by all kinds of useless noise, leading to low damage diagnosis accuracy. To avoid damage misjudgment and leakage, the noise reduction method of vibration response data is required to extract the effective information of structure vibration response. In recent years, scholars have proposed a variety of noise reduction methods to extract the effective information, and achieved effective results, which are mainly divided into the time-domain method, frequency-domain method and time–frequency-domain method.

## Related work

The current research status of the time-domain method, frequency-domain method and time–frequency-domain method are as follows: The effective information of the signal extracted by the time domain method is mainly limited amplitude filtering, arithmetic mean filtering and median filtering method. Among them, Wang et al. used the amplitude filtering method to extract the peak of signal, and the spectrum estimation algorithm was used to estimate the signal spectrum accurately^5^. Lee et al. used fixed median filter to eliminate the impulse noise^6^. The above time-domain noise reduction method is easy to achieve, but the statistical law of vibration signal are easy to ignore. The time-domain noise reduction method has the defect of blindness, and it just can suppress the pulse noise.

The effective information extraction by the frequency domain method is to transform the data from the time domain to the frequency domain, and set the threshold to realize the separation of noise frequency components to achieve noise reduction. Djurdje et al. proposed the generalization of the time infinite impulse response digital filters^7^. Kacar et al. proposed four new voltage-mode universal biquad filters^8^. Kaur et al. proposed the evaluation of fractional order high pass filter based on Operational Transresistance Amplifier (OTRA) using three fractional elements^9^. Brandt et al. used a spectral approach to analyze the characteristics of the filter and the distortions in the processed spectra^10^. The principle of the above frequency domain noise reduction method is intuitive and simple, but for the hydraulic concrete structure vibration signal, this method can just filter the part outside the interference signal, and the band noise cannot be removed.

The time–frequency domain method to extract the effective information is a method to analyze the vibration response in the time–frequency domain, and the sampling signal is converted into the two-dimensional space to analyze. The noise reduction is carried out by comprehensively analyzing the time domain and frequency domain characteristics of the signal^11^. Therefore, this method is more widely used in signal processing. Donoho studied the selection method of wavelet threshold, and a uniform threshold definition method was proposed^12^. Donoho utilized the Stein unbiased estimation method to solve the threshold, and it can improve the noise reduction accuracy^13^. Guo et al. proposed a compound fault diagnosis method of rolling bearing based on wavelet scattering transform and improved soft threshold denoising algorithm^14^. Ding et al. mined the microseismic time series data integrated classification based on improved wavelet decomposition and ELM^15^. The wavelet basis function should be set in advance and cannot be changed after setting, which affects the processing effect of different scales and the adaptive decomposition of signals cannot be realized. The empirical mode decomposition (EMD) method is a time–frequency signal analysis algorithm proposed by Huang et al. It can decompose the signal into a series of inherent mode functions adaptively, and it has a strong non-stationary signal processing ability^16^. Ayenu et al. utilized the mutual information method to select the optimal mode component combination by calculating the correlation coefficient between the original noise response signal and its decomposed intrinsic mode components^17,18^. The noise reduction method based on the EMD algorithm overcomes the difficulty of selecting wavelet base function while retaining the advantage of wavelet multiple resolution, but the theoretical basis of this method is not sufficient, and there may be a modal overlap question caused by the data break point in the decomposition process. The Variational mode decomposition (VMD) is a new signal decomposition algorithm proposed by Dragomiretskiy et al^19^. Because the VMD algorithm has better noise robustness and avoids the modal aliasing problem, it is widely used in non-stationary time series analysis with high complexity and a strong degree of nonlinearity. Ma et al. introduced multiscale sample entropy combined VMD to realize feature extraction^20^. Cheng et al. used the decomposition-based denoising method SSA-VMD to deal with the acoustic emission signal^21^. Wang et al. proposed an adaptive variational mode decomposition technique with differential evolution algorithm^22^.

The number of sensors installed on hydraulic concrete structures is generally large and the amount of response data is large. However, the above noise reduction methods are mostly based on single sensor data, which takes a long time and cannot meet the demand of online damage diagnosis for algorithm operation speed. It is necessary to study the multi-sensor data synchronization noise reduction technology to improve the noise reduction effect.

**Fig. 1:**
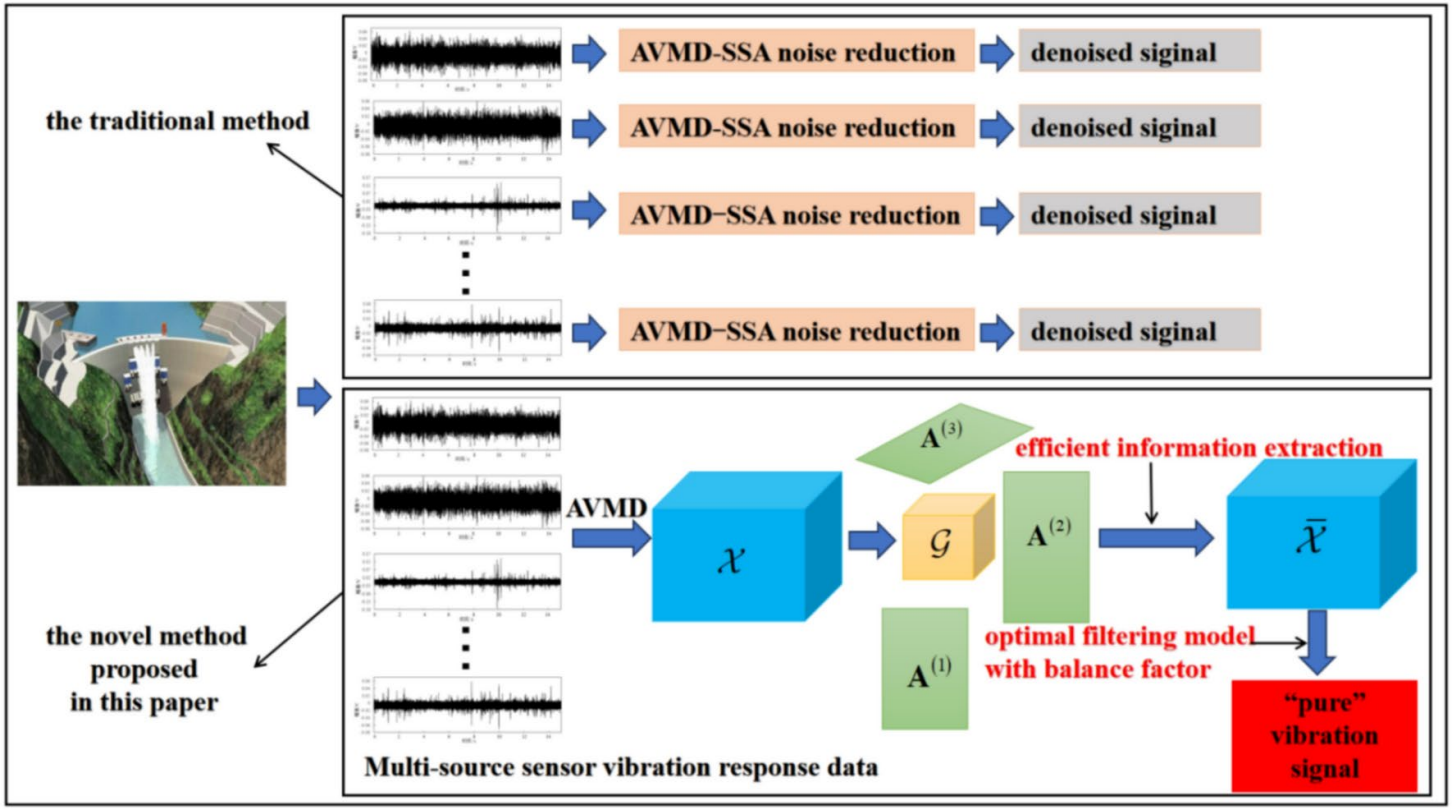
The flowchart of the traditional method and the proposed method.

In this paper, the AVMD algorithm combined tensor analysis tool was used to convert multi-sensor vibration response data into spatio-temporal tensor. A novel tensor efficient information extraction method based on fast orthogonal Tucker factor updating was proposed, and a novel optimal filtering model with balance factor combined the dynamic time programming theory with time weight modification and curvature smoothing algorithm was constructed to reconstruct the effective information extracted above. Thus, the full frequency domain adaptive synchronous noise reduction of three-dimensional spatio-temporal tensor multi-point data was realized. As shown in Fig. 1, since the tensor tool was introduced in this paper, the spatiotemporal correlation between different sensor data was considered in the process of noise reduction.

The paper’s structure is as follows: the content of the methodology is divided into three parts. The first part is the method of converting multi-source sensor signals into three-dimensional space–time tensor. In the second part, on the basis of tensor tools, a novel method of tensor effective information extraction was proposed. In the third part, a new optimal filter model with balance factor was constructed, which can reconstruct the above valid information and realize the simultaneous noise reduction of multi-source data. Then, the vibration response data of intact condition and different damage conditions collected by sluice model were used to verify the rationality of the proposed method and its advantages over other methods.

## Methodology

The hydraulic structure system is an open, dissipative, and complex nonlinear dynamical system, with at least one part or more parts or all parts of the system’s nonlinear characteristics, and there are nonlinear interactions between the different parts. The service condition of hydraulic concrete structures is influenced by environmental factors such as temperature, water temperature and water level. The feature of “open” is mainly manifested as the coupling effect field of multiphase environmental factors. In the nonlinear dynamic system, the essence of nonlinear is the interaction between variables, and the effective information extraction method without considering the correlation between different measuring points can cause over-denoise or under-denoise problems, hence the accuracy of effective information mining is low. In addition, the vast majority of useless noise is randomly mixed with the alternating current interference, the non-Gaussian noise and the small probability noise, hence the noise is random, irregular and without priori. Therefore, it is necessary to study the synchronous noise reduction algorithm of multiple measurement vibration response data to improve the noise reduction efficiency, accuracy and adaptability. In this paper, the improved Adaptive Variational Mode Decomposition (AVMD) method^23^ is used to convert the two-dimensional multi-measurement point vibration response data into a three-dimensional multi-scale space–time tensor. The space–time tensor factor filter sets with fast orthogonal Tucker factor updating based on the Crank–Nicholson-Like criterion is proposed to reduce the noise of the signal initially. The time weight modified Dynamic Time Warping theory and the curvature smoothing algorithm combination model with a balance factor is constructed to realize the effective information mining of vibration response.

### The space–time tensor construction method

**Fig. 2:**
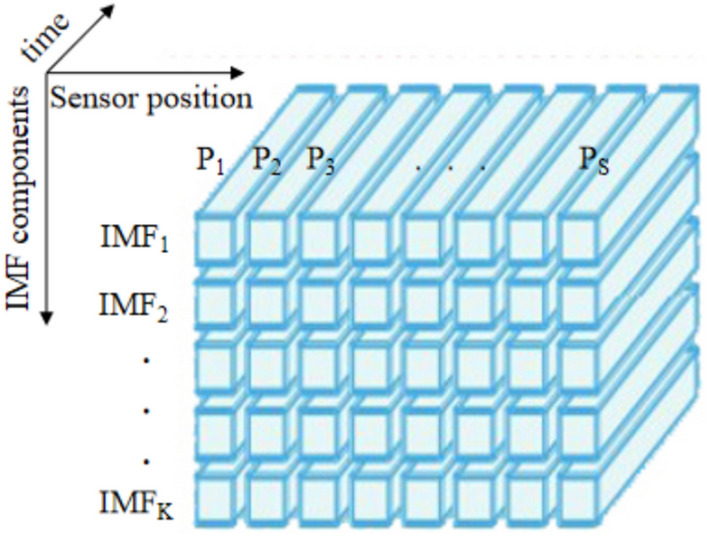
The 3D spatiotemporal tensor.

The vibration response data of the multi-measurement point is a two-dimensional matrix, and the improved AVMD method^23^ was used to convert the two-dimensional vibration response data into a three-dimensional multi-scale space–time tensor $\Psi \in {\mathbb{R}}^{S \times K \times t}$ as shown in Fig. 2. The tensor is a three-dimensional tensor, the first dimension is S, representing the number of sensors; the second dimension is K, representing the number of IMF components in which the data is decomposed; and the third dimension represents the time.

### The preliminary noise reduction method of multiple measuring vibration response

In this paper, the tensor analysis theory is used to analyze the 3-dimensional space–time tensor. One of the most common processing method of tensor analysis is tensor decomposition, but the numerical implementation of tensor decomposition is extremely difficult. The conventional alternative method is the low-rank approximation to realize the approximate decomposition of tensor^24^. The approximate decomposition methods of the tensor are the Canonical Polyadic (CP) decomposition, the Tucker decomposition, the tensor ring decomposition and the tensor chain decomposition and so on. Specifically, the Tucker decomposition method can express the tensor equivalence as a core tensor and three factor matrices. In this paper, the Tucker decomposition method is introduced to reduce the noise of the space–time tensor constructed by the multiple vibration response. In addition, to further improve the computational efficiency and approximation accuracy of Tucker decomposition, the Crank–Nicholson-Like criterion is used to construct the fast orthogonal Tucker factor update method to realize the iterative solution of the core tensor and the three factor matrix, and then the factor matrix is constructed as a factor matrix filter group for noise reduction.

### The factor matrix update method based on the Tucker decomposition

The Tucker decomposition based best low-rank approximation of the space–time tensor ${\mathcal{X}} \in {\mathbb{R}}^{{I_{1} \times I_{2} \times \cdots I_{N} }}$ is a product of a core tensor ${\mathcal{G}}$ and a series of factor matrices ${\mathbf{A}}^{\left( 1 \right)} ,{\mathbf{A}}^{\left( 2 \right)} , \cdots ,{\mathbf{A}}^{\left( N \right)}$

1 $${\mathcal{X}} \approx \left[ {{\mathcal{G};}{\mathbf{A}}^{\left( 1 \right)} ,{\mathbf{A}}^{\left( 2 \right)} , \cdots ,{\mathbf{A}}^{\left( N \right)} } \right]{ = }{\mathcal{G}} \times_{1} {\mathbf{A}}^{\left( 1 \right)} \times_{2} {\mathbf{A}}^{\left( 2 \right)} \cdots \times_{N} {\mathbf{A}}^{\left( N \right)}$$

where N represents the dimension of the tensor, Hence the Tucker decomposition can be converted into an optimization problem as follows

2 $$\begin{aligned} & \mathop {\min }\limits_{{{\mathcal{G}},{\mathbf{A}}^{\left( 1 \right)} ,{\mathbf{A}}^{\left( 2 \right)} , \cdots ,{\mathbf{A}}^{\left( N \right)} }} \left\| {\mathcal{X} - \left[ {{\mathcal{G}};{\mathbf{A}}^{\left( 1 \right)} ,{\mathbf{A}}^{\left( 2 \right)} , \cdots ,{\mathbf{A}}^{\left( N \right)} } \right]} \right\|_{F}^{2} \\ & s.t.\; {\mathcal{G}} \in \mathbb{R}^{{J_{1} \times J_{2} \times \cdots J_{N} }} \\ & {\mathbf{A}}^{\left( n \right)} \in \mathbb{R}^{{I_{n} \times J_{n} }} ,{\mathbf{A}}^{\left( n \right)} {\mathbf{A}}^{\left( n \right)T} = {\mathbf{I}},n = 1,2, \cdots ,N \\ \end{aligned}$$

Since the core tensor ${\mathcal{G}}$ can be obtained directly by the product of the original tensor ${\mathcal{X}}$ and the factor matrix ${\mathbf{A}}^{\left( n \right)}$, the core tensor can be expressed as

3 $${\mathcal{G}} = {\mathcal{X}} \times_{1} {\mathbf{A}}^{\left( 1 \right)T} \times_{2} {\mathbf{A}}^{\left( 2 \right)T} \cdots \times_{N} {\mathbf{A}}^{\left( N \right)T}$$

The update rule for ${\mathbf{A}}^{\left( n \right)}$ is^25^

4 $$\begin{gathered} {\mathbf{A}}^{\left( n \right)} \leftarrow \left( {{\mathbf{I}}_{{R_{n} }} - \eta \left( {{\mathbf{I}}_{{R_{n} }} + \frac{\eta }{2}{\mathbf{B}}^{\left( n \right)} } \right)^{ - 1} {\mathbf{B}}^{\left( n \right)} } \right){\mathbf{A}}^{\left( n \right)} \hfill \\ \, = - {\mathbf{A}}^{\left( n \right)} + \left( {2{\mathbf{A}}^{\left( n \right)} - \eta {\mathbf{G}}^{\left( n \right)} } \right)\left( {{\mathbf{I}}_{{R_{n} }} + \frac{{\eta^{2} }}{4}{{\varvec{\Gamma}}}^{\left( n \right)} } \right)^{ - 1} \hfill \\ \end{gathered}$$

where ${\mathbf{I}}_{{R_{n} }}$ is a matrix of size $R_{n} \times R_{n}$,the step length $\eta > 0$,${\mathbf{B}}^{\left( n \right)} = {\mathbf{G}}^{\left( n \right)} {\mathbf{A}}^{\left( n \right)T} - {\mathbf{A}}^{\left( n \right)T} {\mathbf{G}}^{\left( n \right)T}$,${\mathbf{G}}^{\left( n \right)} = - {\mathbf{C}}^{\left( n \right)} {\mathbf{A}}^{\left( n \right)} - {\mathbf{A}}^{\left( n \right)} {{\varvec{\Lambda}}}^{\left( n \right)}$,${\mathbf{C}}^{\left( n \right)} = {\mathbf{X}}_{\left( n \right)} \left( { \otimes_{k \ne n} {\mathbf{A}}^{\left( k \right)} {\mathbf{A}}^{\left( k \right)T} } \right){\mathbf{X}}_{\left( n \right)}^{T}$,${\mathbf{X}}_{\left( n \right)}$ is n-modular expansion of the tensor ${\mathcal{X}}$,${{\varvec{\Lambda}}}^{\left( n \right)} = - {\mathbf{A}}^{\left( n \right)T} {\mathbf{C}}^{\left( n \right)} {\mathbf{A}}^{\left( n \right)}$,${{\varvec{\Gamma}}}^{\left( n \right)} = {\mathbf{G}}^{\left( n \right)T} {\mathbf{G}}^{\left( n \right)}$.

The step length $\eta$ of the k-th iteration in Eq. (4) is defined by the Barzilai-Borwein method^26,27^

5 $$\eta_{k} = \frac{{{\mathbf{s}}_{k - 1}^{T} {\mathbf{s}}_{k - 1} }}{{{\mathbf{s}}_{k - 1}^{T} {\mathbf{x}}_{k - 1} }}$$

where ${\mathbf{s}}_{k - 1} = vec\left( {{\mathbf{A}}^{\left( n \right)\left( k \right)} - {\mathbf{A}}^{{\left( n \right)\left( {k - 1} \right)}} } \right) = - \eta_{k - 1} vec\left( {{\mathbf{G}}^{{\left( n \right)\left( {k - 1} \right)}} } \right),{\mathbf{x}}_{k - 1} = vec\left( {{\mathbf{G}}^{\left( n \right)\left( k \right)} - {\mathbf{G}}^{{\left( n \right)\left( {k - 1} \right)}} } \right).$ The factor matrix ${\mathbf{A}}^{\left( n \right)}$ is updated iteratively in an internal loop with few iteration times can improve the operation speed of the algorithm.

### The factor matrix filter sets construction method

The relationship between the hydraulic structure vibration response ${\mathcal{X}} \in {\mathbb{R}}^{{K \times S \times \tilde{T}}}$ and the random useless noise ${\mathcal{N}}$ without any prior is complex. The relationship is generally difficult to express explicitly. Without loss of generality, the relationship is expressed as follows

6 $${\mathcal{X}} = \overline{x} + {\mathcal{N}}$$

where ${\overline{\mathcal{X}}}$ is the vibration response signal tensor after noise reduction. The low-rank approximation of ${\mathcal{X}}$ is as follows

7 $${\mathcal{X}} \approx {\mathcal{M}}{ = }{\mathcal{G}} \times_{1} {\mathbf{A}}^{\left( 1 \right)} \times_{2} {\mathbf{A}}^{\left( 2 \right)} \times_{3} {\mathbf{A}}^{\left( 3 \right)}$$

where, ${\mathcal{M}}$ is the best low-rank approximation tensor of ${\mathcal{X}}$, ${\mathcal{G}} \in {\mathbb{R}}^{P \times Q \times R}$ is the core tensor and ${\mathbf{A}}^{\left( n \right)} \left( {n = 1,2,3} \right)$ is the factor matrix. The factor matrix is obtained by the fast orthogonal Tucker factor update method. The factor matrix ${\mathbf{A}}^{\left( n \right)} \left( {n = 1,2,3} \right)$ can be regarded as 1-mode, 2-mode and 3-mode filters. To achieve noise reduction of the data, it is important to construct the appropriate factor filter sets. Since the Singular Value Decomposition (SVD) method is an effective signal noise reduction tool^28^, in this paper, the SVD with an appropriate threshold k is used to decompose the factor matrix ${\mathbf{A}}^{\left( n \right)} \left( {n = 1,2,3} \right)$, and the factor filter sets are constructed by the construction of factor matrix. Take ${\mathbf{A}}^{\left( 1 \right)} \in {\mathbb{R}}^{K \times P}$ for example, the matrix can be decomposed into

8 $${\mathbf{A}}^{\left( 1 \right)} = {\mathbf{U\Sigma V}}^{T}$$

where ${\mathbf{U}} = \left[ {{\mathbf{u}}_{1} ,{\mathbf{u}}_{2} , \cdots ,{\mathbf{u}}_{K} } \right] \in {\mathbb{R}}^{K \times K} ,{\mathbf{V}} = \left[ {{\mathbf{v}}_{1} ,{\mathbf{v}}_{2} , \cdots ,{\mathbf{v}}_{P} } \right] \in {\mathbb{R}}^{P \times P}$.${{\varvec{\Sigma}}}$ is the singular values of ${\mathbf{A}}^{\left( 1 \right)}$ arranged in descending order, and

9 $${{\varvec{\Sigma}}} = \left[ {diag\left( {\sigma_{1} ,\sigma_{2} , \cdots ,\sigma_{l} } \right),{\mathbf{0}}} \right] \in {\mathbb{R}}^{K \times P} ,$$

where $l = \min \left( {K,P} \right),\sigma_{1} \ge \sigma_{2} \ge \cdots \ge \sigma_{l}$. Since the vibration response data tensor ${\mathcal{X}}$ consists the effective information and the useless noise, the factor matrix generated by the low-rank approximation algorithm is also composed of the effective information and the useless noise. Singular value decomposition of the factor matrix, the singular value can reflect the energy distribution of effective information and the useless noise, the first k large singular values reflect the main energy of the signal, that is, the useful information, and the small singular value reflects the noise, take the singular value of noise to zero can realize noise reduction.

The mutant points often carry important information relative to smoothly changing points. Therefore, in the sequence of singular values, the maximum mutation point represents the boundary between effective information and the useless noise, indicating that there is the maximum difference between the singular value before and after this position. The fundamental reason for this difference is that the energy distribution of useful signal and noise is very different, which shows the phenomenon on the different singular value. Therefore, the mutation of singular value is the distinction point between useful signal and noise. The singular value difference spectrum is constructed to find the mutation position of singular value, and the index number of the mutation position is the threshold k. Specifically, $b_{i} = \sigma_{i} - \sigma_{i + 1} { ,}\left( {i = 1,2, \cdots ,l - 1} \right)$,and the vector $B = \left[ {b_{1} ,b_{2} , \cdots ,b{}_{l - 1}} \right]$ formed by $b_{i}$ is the singular value difference spectrum. When the difference between two adjacent singular values is large, a peak is generated in the difference spectrum, and there must be a maximum peak $\max (b_{i} ) = b_{k}$ in the entire difference spectrum. According to the definition of the difference spectrum, the singular value sequence has the maximum mutation at k. Therefore, the components corresponding to the first k singular values are the effective components of factor matrix, while the components corresponding to the other singular values after the mutation positions are redundant noise. After the factor matrix reconstruction, the factor matrix filter set can be obtained

10 $${\tilde{\mathbf{A}}}^{\left( 1 \right)} = \sum\limits_{i = 1}^{k} {\sigma_{i} } {\mathbf{u}}_{i} {\mathbf{v}}_{i}$$

In a similar way, the factor filter set ${\tilde{\mathbf{A}}}^{\left( 2 \right)}$ and ${\tilde{\mathbf{A}}}^{\left( 3 \right)}$ are obtained, and the core tensor ${\mathcal{G}}$ can be updated to ${\tilde{\mathcal{G}}}$

11 $$\widetilde{{\mathcal{G}}} = {\mathcal{X}} \times_{1} \tilde{{\mathbf{A}}}^{\left( 1 \right)T} \times_{2} \tilde{{\mathbf{A}}}^{\left( 2 \right)T} \times_{3} \tilde{{\mathbf{A}}}^{\left( 3 \right)T}$$

According to the Eq. (7), the signal tensor after noise reduction can be obtained to achieve the initial signal noise reduction

12 $$\overline{{\mathcal{X}}} \approx \widehat{{\overline{{\mathcal{X}}} }} = \widetilde{{\mathcal{G}}} \times_{1} \tilde{{\mathbf{A}}}^{\left( 1 \right)} \times_{2} \tilde{{\mathbf{A}}}^{\left( 2 \right)} \times_{3} \tilde{{\mathbf{A}}}^{\left( 3 \right)}$$

where ${\overline{\mathcal{X}}}$ is the signal tensor after initial noise reduction, and ${\hat{\overline{\mathcal{X}}}}$ is the best low-rank approximation of the signal tensor after initial noise reduction.

### The full-frequency domain adaptive secondary noise reduction method

The method in Sect. 2.2 can be used to reduce the signal noise initially, but the full- frequency domain noise mixed in the signal is not eliminated, and the frequency composition of the full-frequency domain noise is unknown, random and complex. It is essential to find a method for adaptive secondary noise reduction in the full frequency domain after the initial noise reduction, hence the IMF component of each side slice in the spatio-temporal tensor can be adaptively reconstructed to obtain the pure multi-measurement point vibration response data. When reconstructing each side slice, an appropriate method needs to be selected to screen the effective IMF component. A high-pass, band-pass and low-pass filter can be obtained by selecting and combining different IMF components. Assuming that the decomposition order is $i = 1,2, \cdots ,n$, hence the high-pass filter can be represented as

13 $${\text{HF}}_{e} = \sum\limits_{i = 1}^{e} {{\text{IMF}}_{i} } \left( t \right)$$

The bandpass filter can be expressed as

14 $${\text{BF}}_{f}^{g} = \sum\limits_{i = f}^{i = g} {{\text{IMF}}_{i} } \left( t \right)$$

The low-pass filter can be expressed as

15 $${\text{LF}}_{h} = \sum\limits_{i = h}^{n} {{\text{IMF}}_{i} } \left( t \right)$$

where $e,f,g,h$ are the filter restriction points of the high-pass, band-pass and low-pass filters, respectively. The filter restriction point can be moved and crossed according to the filtering requirements, and the corresponding filtering effect can be achieved by reconstructing different IMF components.

Since there is no prior knowledge of the useless noise in full frequency domain, it is not known which IMF combination can obtain the best filtering effect. To solve this problem, the similarity and smoothness of the filtered tube fiber reconstruction data and the intact tube fiber reconstruction data are optimized and evaluated. The time weight correction Dynamic Time Warping (DTW) algorithm is introduced for quantitative evaluation of similarity, and the curvature coefficient method is used to evaluate the smoothness. The weight factor of similarity and smoothness is proposed to build the best filtering model, and the model can dynamically adjust the filtering parameters according to the signal characteristics and noise distribution to realize effective signal information extraction.

### The time weight correction dynamic time warping based sequence similarity quantification method

Time series similarity belongs to the content of the curve matching, in which the “distance” is often used to quantify the representation of time series similarity. Among them, the most classical distance measurement is the Euclidean distance^29^, but the shape similarity and the trend dynamics could not be measured by it^30^. Compared with the Euclidean distance, the Dynamic Time Warping (DTW) can measure the shape similarity and trend dynamics by time series tracks, hence the measurement accuracy of time series similarity is improved. Therefore, the DTW is chosen as the similarity measurement in this paper.

The basic idea of the DTW is to use the dynamic planning strategy to plan two sequences time axes, and then find and determine the optimal correspondence between sequences^31^. Assuming that two time series are $A = \left\{ {a_{1} ,a_{2} , \cdots ,a_{n} } \right\}$ and $B = \left\{ {b_{1} ,b_{2} , \cdots ,b_{m} } \right\}$, the goal of the DTW is to find an optimal path from $\left( {1,1} \right)$ to $\left( {n,m} \right)$ ensuring a minimum cumulative distance of the path. The conventional DTW algorithm calculates the similarity of two time series using equal time weights, but the DTW accumulates the effects of Euclian distances, and the effect of the current moment nearest point may be influenced by the historical point. For online damage diagnosis of hydraulic concrete structure, if the structure is always in health condition, the structure vibration response is stable. However, if the structure has damage, the vibration response of the structure will fluctuate, in the similarity measurement, the current moment near time point is more important than historical. The time effect in similarity measurement of the conventional DTW can be considered, and a larger weight is given to the nearest time point, a small weight is given to the historical time point. The European distance between different points with different time weight are used to construct the cumulative distance, and the time weight in the planning matrix $\left( {i,j} \right)$ is^32^

16 $${\mathbf{W}}\left( {i,j} \right) = \frac{{\sqrt {\left( {{i \mathord{\left/ {\vphantom {i n}} \right. \kern-0pt} n}} \right)^{2} + \left( {{j \mathord{\left/ {\vphantom {j m}} \right. \kern-0pt} m}} \right)^{2} } }}{\sqrt 2 },\left( {i = 1,2, \cdots ,n;j = 1,2, \cdots ,m} \right)$$

where ${\mathbf{W}}\left( {i,j} \right) \in \left[ {0,1} \right]$. $x\left( i \right) = \frac{i}{n}$ and $y\left( j \right) = \frac{j}{m}$ represent the i-th and j-th sample importance of sequences A and B, respectively.

The dynamic programming state transfer equation after the time-weight modification can be written as

17 $$\left\{ \begin{gathered} {\mathbf{D}}\left( {1,1} \right) = {\tilde{\mathbf{W}}}\left( {1,1} \right) * d\left( {1,1} \right) \hfill \\ {\mathbf{D}}\left( {1,j} \right) = {\tilde{\mathbf{W}}}\left( {1,j} \right) * d\left( {1,j} \right) + {\mathbf{D}}\left( {1,j - 1} \right) \hfill \\ {\mathbf{D}}\left( {i,1} \right) = {\tilde{\mathbf{W}}}\left( {i,1} \right) * d\left( {i,1} \right) + {\mathbf{D}}\left( {i - 1,1} \right) \hfill \\ {\mathbf{D}}\left( {i,j} \right) = {\tilde{\mathbf{W}}}\left( {i - 1,j - 1} \right) * d\left( {i - 1,j - 1} \right) \hfill \\ \, + \min \left[ {{\mathbf{D}}\left( {i,j - 1} \right),{\mathbf{D}}\left( {i - 1,j} \right),{\mathbf{D}}\left( {i - 1,j - 1} \right)} \right] \hfill \\ \end{gathered} \right.$$

where ${\tilde{\mathbf{W}}}\left( {i,j} \right)$ is the result of inverting each column of the weight matrix ${\mathbf{W}}\left( {i,j} \right)$,$d\left( {i,j} \right)$ is the Euclidean distance between each two points $a_{i}$ and $b_{j}$,$d\left( {i,j} \right) = \sqrt {\left( {a_{i} - b_{j} } \right)^{2} } \, \left( {i = 1 \, , \, 2 \, , \, \cdots \, , \, n{ ; }j = 1 \, , \, 2 \, , \, \cdots \, , \, m} \right)$.${\mathbf{D}}\left( {i,j} \right)$ is the minimum cumulative Euclidean distance from $d(1,1)$ to $d(i,j)$.The optimal distance over the planning path can be added to obtain the sequence similarity corrected by time weight

18 $${\text{DTW}}\left( {A,B} \right){ = }\sum\nolimits_{1}^{k} {{\tilde{\text{W}}}\left( {i,j} \right)*d(i,j)} \, (d(i,j) \in \tilde{P})$$

where $\tilde{P} = \left\{ {\tilde{p}_{1} ,\tilde{p}_{2} \cdots ,\tilde{p}_{{\tilde{k}}} } \right\}$ is the planning path represents the matching relationship of each point in sequences A and B back-traced after time weight correction,$\max (n,m) \le k \le n + m$.

### The curvature-based sequence smoothness quantization method

The noise is often random and mutated, and an excellent noise reduction algorithm also meets the sequence smoothness. Therefore, the curvature coefficient is used to quantify the sequence smoothness. Suppose two curves $$C_{1} \left( {x_{1} } \right)$$ and $$C_{2} \left( {x_{2} } \right)$$$$\left( {x_{1} ,x_{2} \in \left[ {0,n} \right]} \right)$$, and if the two curves have equal curvature at the point $$t$$,then

19 $$K_{{C_{1} \left( t \right)}} = \frac{{C^{\prime\prime}_{1} \left( t \right)}}{{\left[ {1 + C^{\prime}_{1} \left( t \right)} \right]^{{{\raise0.7ex\hbox{$3$} \!\mathord{\left/ {\vphantom {3 2}}\right.\kern-0pt} \!\lower0.7ex\hbox{$2$}}}} }} = \frac{{C^{\prime\prime}_{2} \left( t \right)}}{{\left[ {1 + C^{\prime}_{2} \left( t \right)} \right]^{{{\raise0.7ex\hbox{$3$} \!\mathord{\left/ {\vphantom {3 2}}\right.\kern-0pt} \!\lower0.7ex\hbox{$2$}}}} }} = K_{{C_{2} \left( t \right)}}$$

The Eq. (19) can be got

20 $$\left\{ \begin{gathered} {{C^{\prime\prime}_{1} \approx \left[ {C_{1} \left( {1 - 2h} \right) - 2C_{1} \left( {1 - h} \right) + C_{1} \left( t \right)} \right]} \mathord{\left/ {\vphantom {{C^{\prime\prime}_{1} \approx \left[ {C_{1} \left( {1 - 2h} \right) - 2C_{1} \left( {1 - h} \right) + C_{1} \left( t \right)} \right]} {h^{2} }}} \right. \kern-0pt} {h^{2} }} \hfill \\ {{C^{\prime\prime}_{2} \approx \left[ {C_{2} \left( {0 + 2h} \right) - 2C_{2} \left( {0 + h} \right) + C_{2} \left( t \right)} \right]} \mathord{\left/ {\vphantom {{C^{\prime\prime}_{2} \approx \left[ {C_{2} \left( {0 + 2h} \right) - 2C_{2} \left( {0 + h} \right) + C_{2} \left( t \right)} \right]} {h^{2} }}} \right. \kern-0pt} {h^{2} }} \hfill \\ \end{gathered} \right.$$

where *h* is the unit step size. According to the above equation and the principle of the curve can be derivable from left to right, the smoothness of the filter curve $f\left( x \right)$ at the point $x = t$ can be defined as follows

21 $$S_{smoothness} \left| {_{x = t} } \right. = f\left( {t + 2h} \right) - f\left( {t - 2h} \right) - 2\left[ {f\left( {t + h} \right) - f\left( {t - h} \right)} \right]$$

**Fig. 3:**
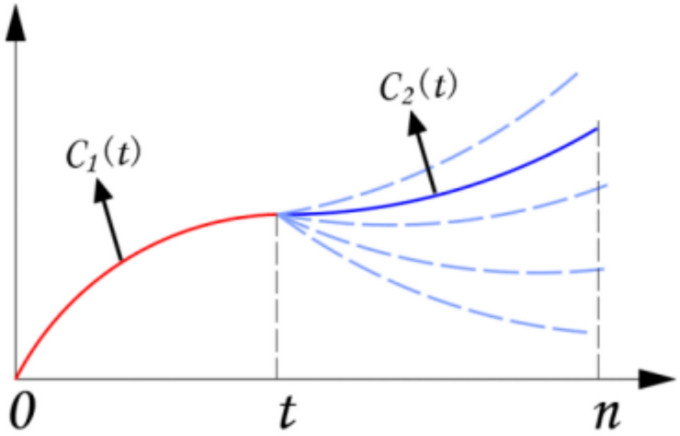
The schematic diagram of the curve smoothness at one point.

As defined in Eq. (21), if the smoothness of the point $x = t$ in the time-domain distribution curve of the sequence is closer to 0, the neighborhood of the point $t$ is smoother, as shown in Fig. 3

For sequences of length n, a smoothness can be calculated for each point in the sequence, and the closer each point is to 0, the smoother the sequence is at that point. Since the root mean square error measures the degree of deviation between two sequences. Therefore, the root mean square error of the calculated smoothness set and the equal length set of element 0 can be defined as the smoothness of the sequence $S_{SM}$

22 $$\begin{gathered} S_{SM} = \sqrt {\frac{1}{n}\sum\limits_{i = 1}^{n} {\left( {S_{smoothness} \left| {_{x = i} } \right. - 0} \right)^{2} } } \hfill \\ { = }\sqrt {\frac{1}{n}\sum\limits_{i = 1}^{n} {\left( {S_{smoothness} \left| {_{x = i} } \right.} \right)^{2} } } \hfill \\ \end{gathered}$$

### Construction method of the optimal filter model

Filter similarity and smoothness are a pair of contradictions. If the similarity between reconstruction signal and original signal is high, the smoothness is low. Otherwise, a low similarity with a high smoothness. To balance this contradiction, a balance factor $\lambda$ is used to ensure that the smoothness and similarity of the reconstruction signal reach a optimal state

23 $${\text{VMD}}_{opt} = \min \left\{ {\lambda * {\text{DTW}}\left( {A,B} \right) + \left( {1 - \lambda } \right) * S_{SM} } \right\}$$

where ${\text{VMD}}_{opt}$ is the optimal filtering effect, $\lambda$ and $1 - \lambda$ is the balance factor of the similarity and smoothness respectively. The IMF component combination of minimum ${\text{VMD}}_{opt}$ value is the optimal filtering result.

**Fig. 4:**
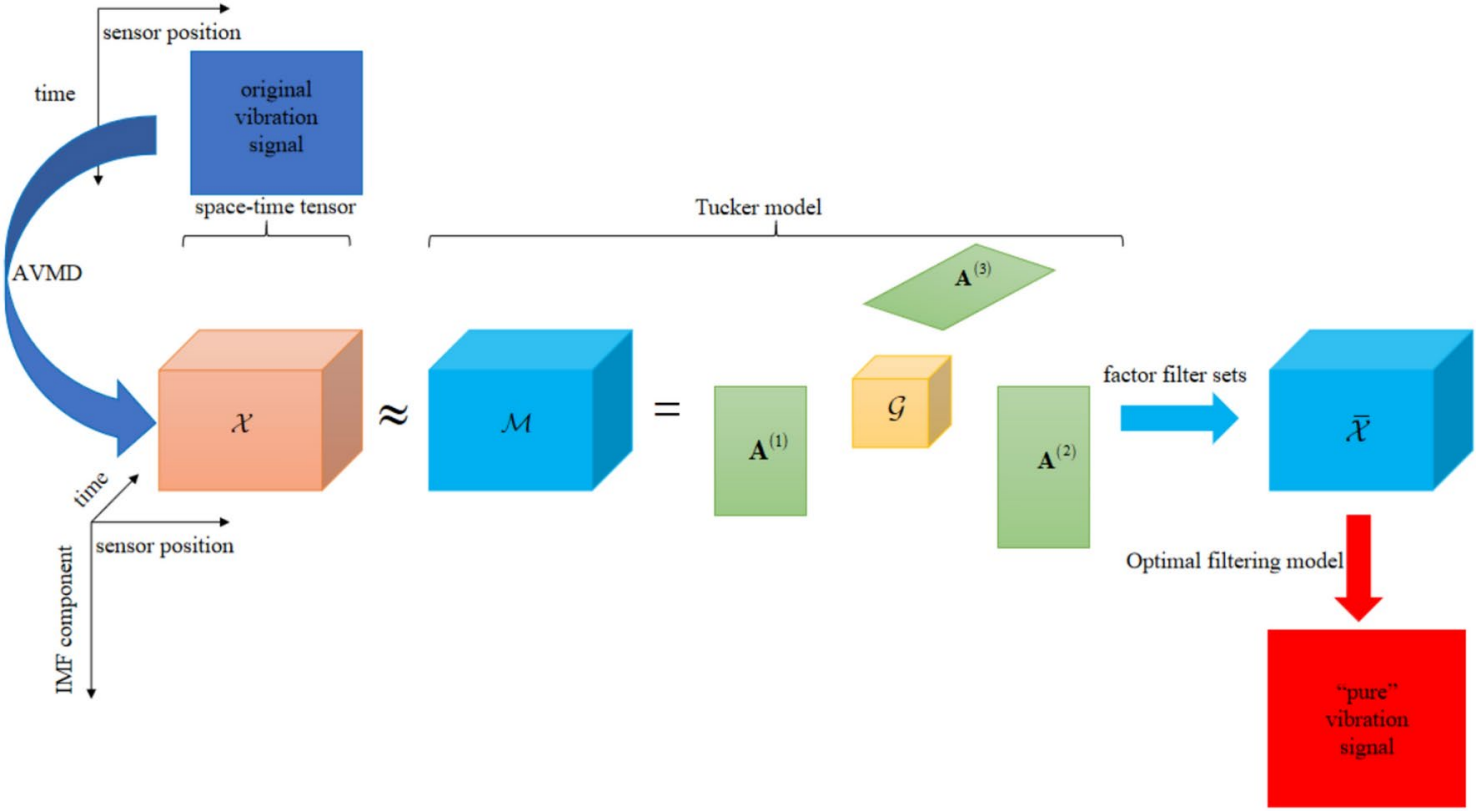
The synchronous adaptive noise reduction method of multi-source vibration data.

In summary, the idea of synchronous noise reduction method of multiple measurement point vibration response data is shown in Fig. 4, and the steps are as follows:

*Step 1*: the AVMD is used to decompose the multiple measurement vibration response signal into IMF component signals of different scales, and the IMF signals are used to construct the spatiotemporal tensor ${\mathcal{X}} \in {\mathbb{R}}^{{K \times S \times \tilde{T}}}$. Where K is the number of IMF component. S is the number of sensors. $\tilde{T}$ is the length of the time window;

*Step 2*: the fast orthogonal Tucker factor update method is used to find the best low-rank approximation tensor ${\mathcal{M}}$ of the tensor ${\mathcal{X}}$.Where the tensor ${\mathcal{M}}$ can be expressed as the product of the core tensor ${\mathcal{G}}$ and the three-factor matrices ${\mathbf{A}}^{\left( 1 \right)} ,{\mathbf{A}}^{\left( 2 \right)}$ and ${\mathbf{A}}^{\left( 3 \right)}$;

*Step 3*: the factor matrix filter set is built to reduce the noise in the tensor initially, and the redundant noise is eliminated to obtain the tensor after the initial noise reduction;

*Step 4*: The optimal filtering model is used to reconstruct the tensor ${\overline{\mathcal{X}}}$ to realize the full frequency domain adaptation, so that the relatively “pure” vibration response data can be obtained, and the effective signal information mining can be realized.

### Example

#### Experimental verification of the sluice model

**Fig. 5:**
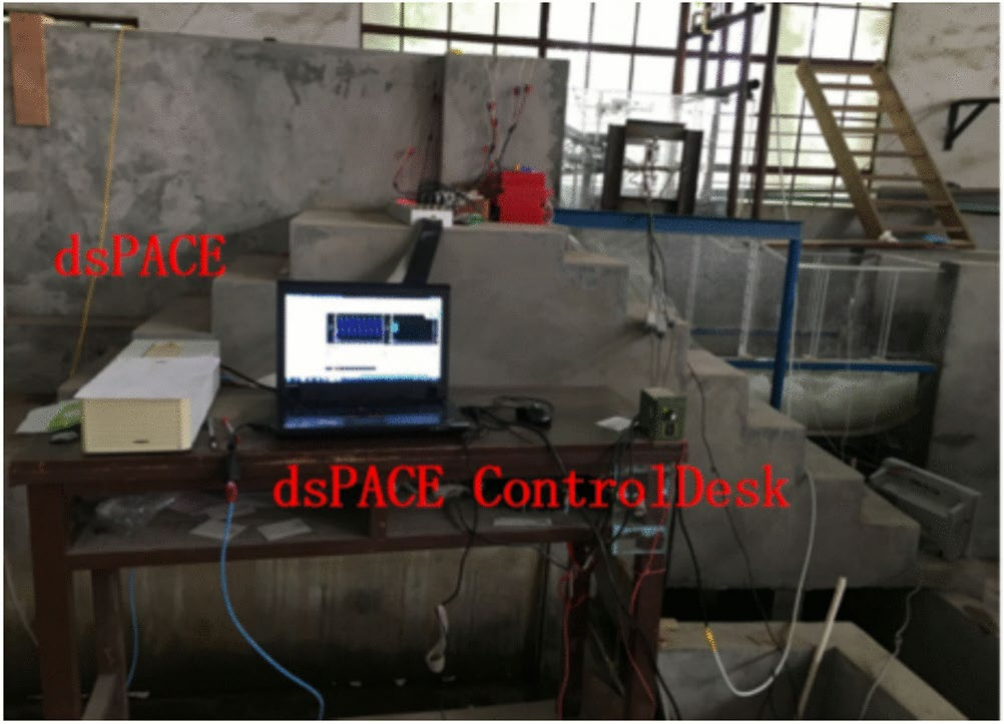
The signal acquisition platform.

**Fig. 6:**
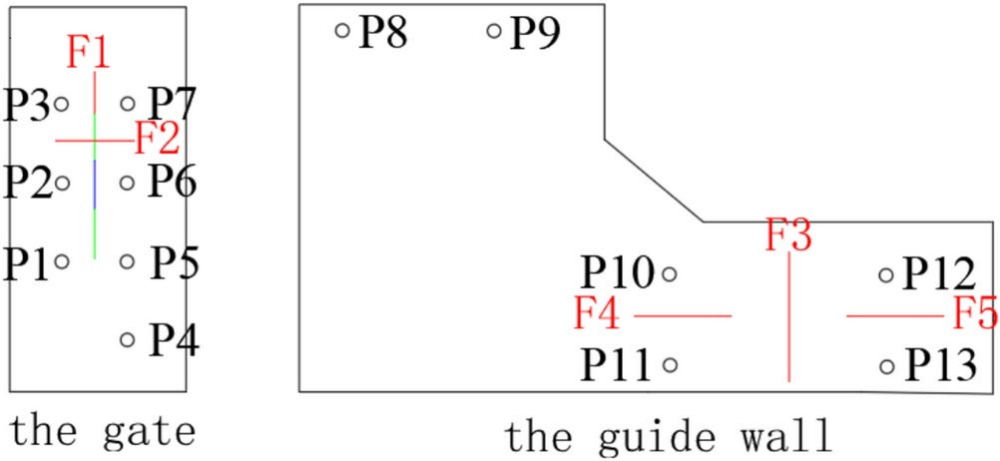
The sensor layout and crack location map.

**Fig. 7:**
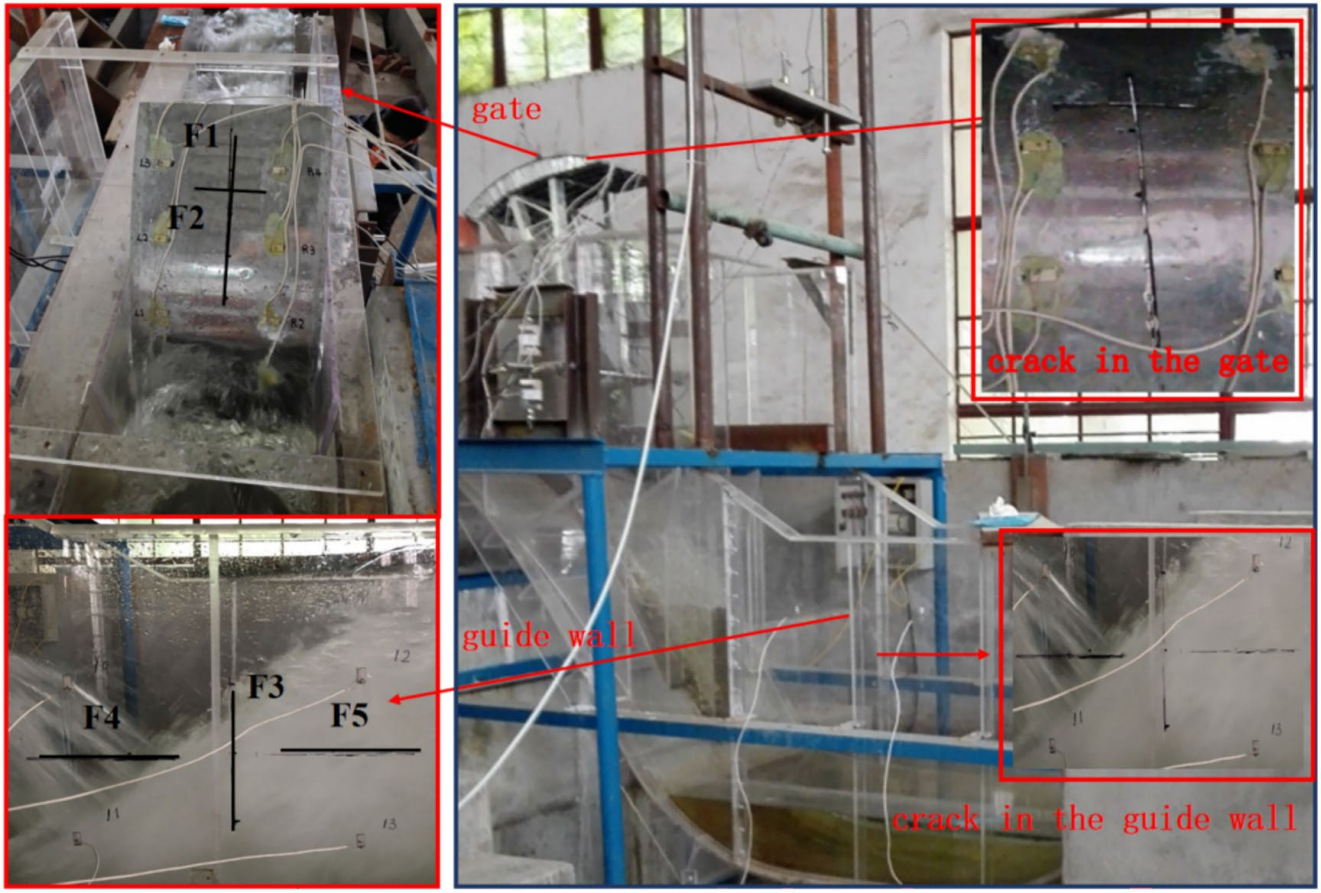
The model photograph of crack location.

To verify the tensor tool considering the spatio-temporal correlation information between different sensors based signal effective information extraction method combined the balance factor model based reconstruction algorithm proposed in this paper can improve the damage diagnosis accuracy of hydraulic concrete structures based on vibration response. In addition, to certificate the proposed method has greater advantages over the traditional single-point-sensor-based AVMD-SSA, EEMD-SVD and EMD-SVD algorithms. A sluice model of 1:45 was established, and the damage was simulated by setting artificial cracks of different lengths and positions. The vibration signals collected in this experiment were used to verify the necessity and advantages of effective information extraction in this paper. The piezoelectric ceramic sensors and instruments used in the experiment are shown in Fig. 5. The gate opening degree was set to 10 cm to realize the discharge excitation. The sensor arrangement and crack position details are shown in Figs. 6 and 7.

### The signal acquisition process

1. When the gate was closed and the water level reached 105 cm, open the gate of 10 cm and discharge the water. The intact condition signal was collected when the discharge is stable, recorded as working condition C1
2. Five damage conditions were designed corresponding to the following cutting process: the cutting machine was used on the gate to get a length of 6 cm longitudinal crack F1 recorded as working conditions C6. The F1 was extended to 12 cm recorded as working condition C11. The F1 was extended to 18 cm recorded as working condition C16. The F1 was extended to 24 cm recorded as working condition C21. A length of 6 cm transverse crack F2 was recorded as working conditions C26. For ease of description, the above five cases were denoted as F1L1, F1L2, F1L3, F1L4, F2

The signal acquisition process of the damage condition was as follows: for the case of F1L1, when the gate was closed and the water level was 105 cm, open the gate of 10 cm and discharge the water. The signal was collected when the discharge is stable. Then measure the vibration response signal when the water level was 110 cm (gate of 15 cm). Similarly, the vibration signals of the other damage conditions are collected.

**Fig. 8:**
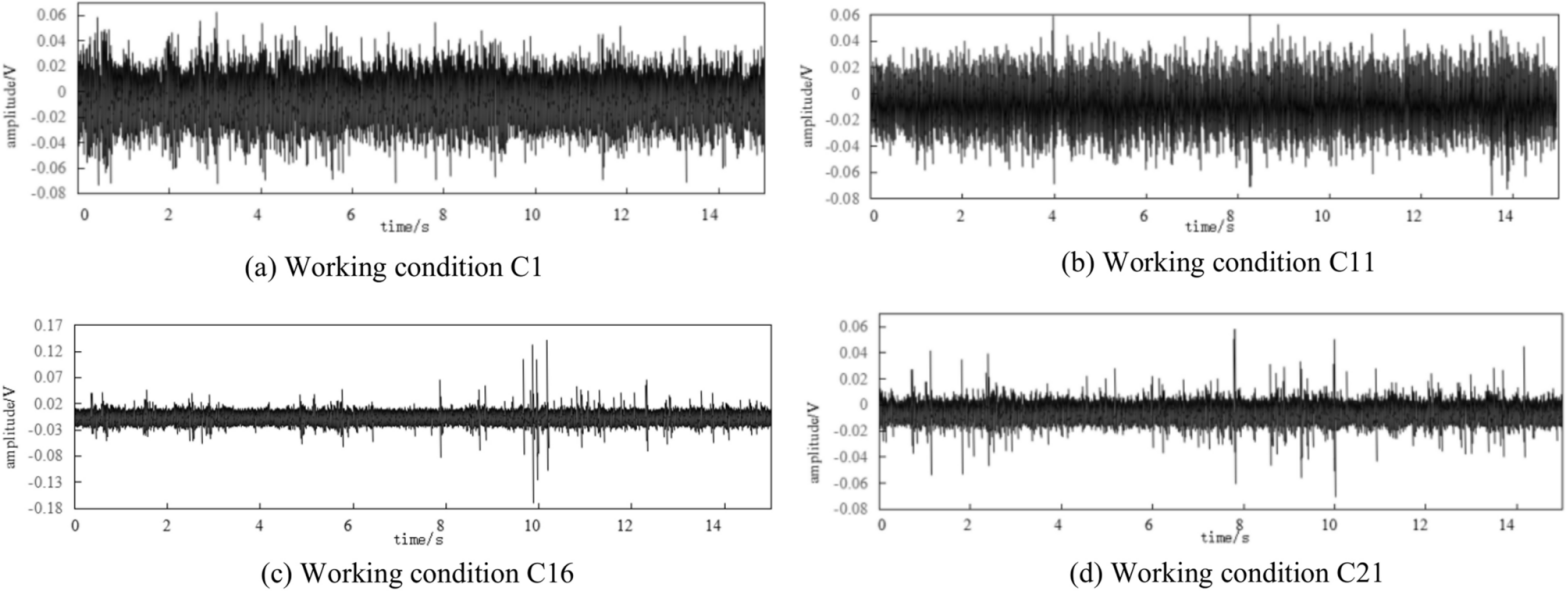
The measured data diagram of P10 under various damage working conditions.

From the collected vibration response signal, the data of point P1 under intact conditions and three damage conditions is shown in Fig. 8. The figures indicated that the time domain distribution of the signal changes significantly in intact and different damage conditions, and the signal amplitude decreases as the degree of structural damage increases.

### Noise reduction of the vibration signal

**Fig. 9:**
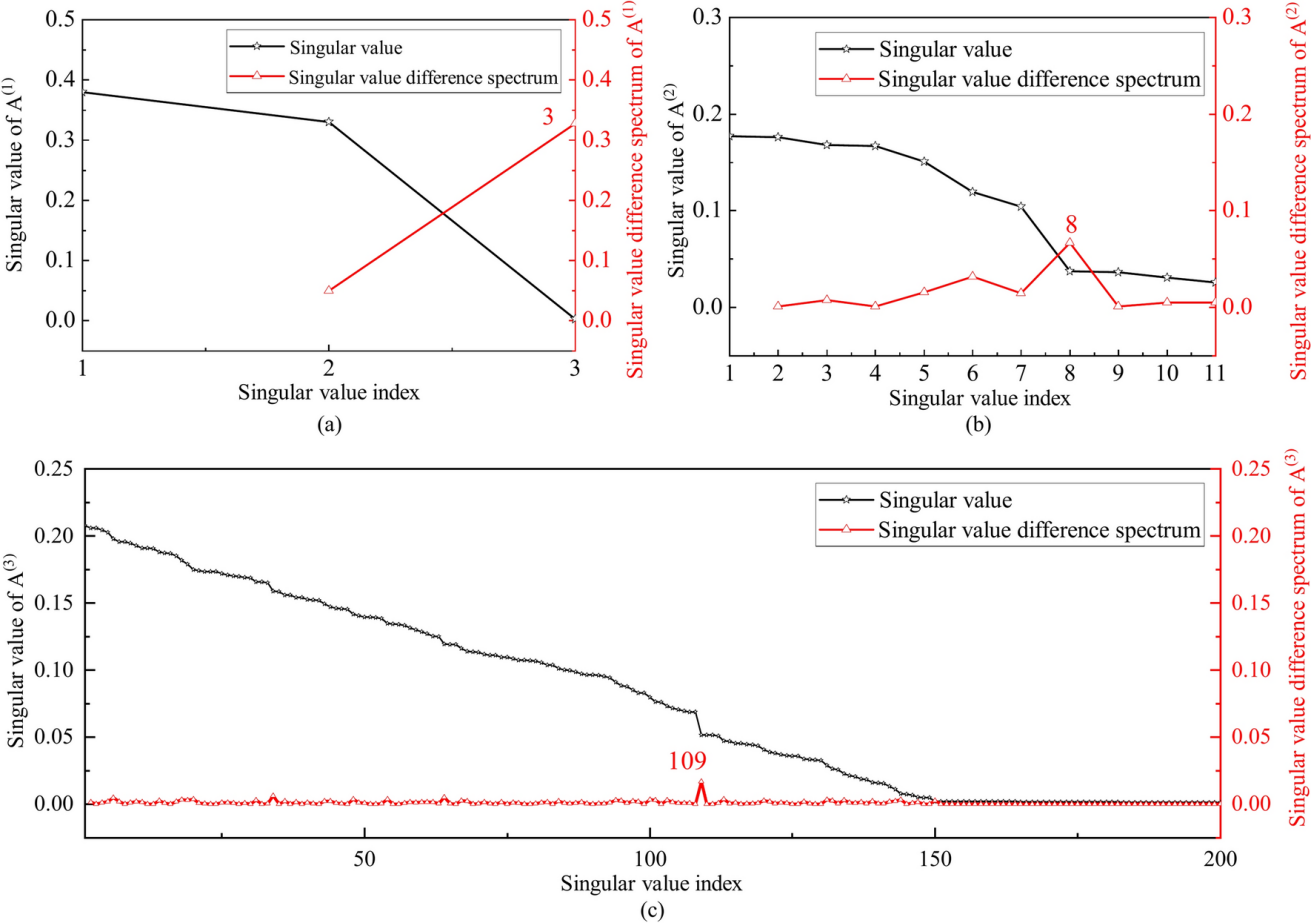
The singular values of factor matrix and their difference spectra.

Taken the condition C1 as an example: the sampling time is 500 s, and the noise reduction sliding time window is 25 s with the number of sampling points 2500. The responses of the 13 sensors were decomposed by the AVMD method synchronously, and by adaptive K value screening, K = 5. A spatiotemporal tensor ${\mathcal{X}} \in {\mathbb{R}}^{{5 \times 13 \times \tilde{T}}} ,\tilde{T} = 2820$ was constructed and updated by the fast orthogonal Tucker factor updating method with the step size of 100. The rank was selected by trial calculation, and the rank $R_{1} = 3,R_{2} = 11,R_{3} = 200$ with the smallest reconstruction loss was selected as the optimal rank. After calculating a core tensor ${\mathcal{G}} \in {\mathbb{R}}^{3 \times 11 \times 200}$ and three factor matrix ${\mathbf{A}}^{\left( 1 \right)} \in {\mathbb{R}}^{5 \times 3} ,{\mathbf{A}}^{\left( 2 \right)} \in {\mathbb{R}}^{13 \times 11} ,{\mathbf{A}}^{\left( 3 \right)} \in {\mathbb{R}}^{1520 \times 200}$ under the available optimal rank are obtained. The singular values of the factor matrix and its difference spectrum are shown in Fig. 9. In the figure, the singular value of ${\mathbf{A}}^{\left( 1 \right)}$ reaches peak value in the difference spectrum at 3, that is, k = 3. The singular value of ${\mathbf{A}}^{\left( 2 \right)}$ reaches peak value in the difference spectrum at 8, that is, k = 8. The singular value of ${\mathbf{A}}^{\left( 3 \right)}$ reaches peak value in the difference spectrum at 109, that is, k = 109.The factor filter set is constructed by Eq. (21) combined with the core tensor ${\mathcal{G}}$, and the tensor ${\overline{\mathcal{X}}} \in {\mathbb{R}}^{{5 \times 13 \times \tilde{T}}} ,\tilde{T} = 2820$ was reconstructed.

Then, the optimal filtering model with balance factor combined the dynamic time programming theory with time weight modification and curvature smoothing algorithm was used to reconstruct the effective information tensor ${\overline{\mathcal{X}}}$ extracted above. At this point, one-time synchronous noise reduction of the data collected by the 13 sensors was achieved, denote as DATA_C1P1_ ~ DATA_C1P13_.

Using the same method, the data of other operating conditions after noise reduction were obtained, denote as DATA_C6P1_ ~ DATA_C6P13_, DATA_C11P1_ ~ DATA_C11P13_, DATA_C16P1_ ~ DATA_C16P13_, DATA_C21P1_ ~ DATA_C21P13_, DATA_C26P1_ ~ DATA_C26P13_, respectively.

### Verification of the proposed method

To verify the vibration data noise reduction algorithm proposed in this paper can improve the damage diagnosis ability of hydraulic concrete structures, the machine learning algorithm XGBoost was used to train and test a classification model on the data before and after de-noising in the non-destructive and different degrees of damage conditions as the training set and the test set. If the accuracy of the test classification results was improved, it indicated that the data differentiation of different damage conditions after noise reduction is greater, which proves that the noise reduction algorithm can improve the accuracy of damage diagnosis.

Taking the sensor P1 on the gate as an example, when the gate opening is 10 cm, the 100 time windows data of length 5 s effective information extracted by the method proposed in this paper under condition C1, C6, C11, C16, C21 and C26, denoted as train_DATA_C1P1_ ~ train_DATA_C26P1_ were chosen to train the XGBoost classification model. The 40 time windows data of length 5 s effective information extracted by the method proposed in this paper under condition C1, C6, C11, C16, C 16, C21 and C26, denoted as test_DATA_C1P1_ ~ test_DATA_C26P1_ were chosen to test the XGBoost classification model. The test results are shown in Table 1.

**Table 1:** Classification and testing indicators of the effective information extracted by the proposed method under different working conditions.

Operating mode	The method of this paper		
	Accuracy rate	recall	F1 metric
C1	0.9744	1.0000	0.9870
C6	0.8710	1.0000	0.9310
C11	1.0000	0.9375	0.9677
C16	0.8721	0.8065	0.8333
C21	0.8649	0.9697	0.9143
C26	0.9412	0.8205	0.8767
Precision		0.9200	

The original signal without noise reduction of the sensor P1 under condition C1, C6, C11, C16, C21 and C26, denoted as train_ORIDATA_C1P1_ ~ train_ORIDATA_C26P1_ were chosen to train the XGBoost classification model. The 40 time windows data of length 5 s original signal without noise reduction of the sensor P1 under condition C1, C6, C11, C16, C21 and C26, denoted as test_ORIDATA_C1P1_ ~ test_ORIDATA_C26P1_ were chosen to test the XGBoost classification model. The test results are shown in Table 2.

**Table 2:** Classification and testing indicators of the original signal without noise reduction under different working conditions.

Operating mode	original signal without noise reduction		
	Accuracy rate	Recall	F1 metric
C1	0.9500	1.0000	0.9744
C6	0.6789	0.7037	0.6909
C11	0.7200	0.5625	0.6316
C16	0.6154	0.7742	0.6857
C21	0.6053	0.6970	0.6479
C26	0.5667	0.4359	0.4928
Precision		0.6950	

The data results in Tables 1 and 2 show that if the noise reduction method proposed in this paper is used, the classification and test accuracy of data collected under non-destructive and different damage conditions can be improved from 0.6950 to 0.9200, and the differentiation of data under different damage conditions is greater. Therefore, using the data after noise reduction for damage diagnosis, the accuracy will be higher.

To verify the proposed algorithm has more advantages than the traditional noise reduction algorithm, the traditional single point based AVMD-SSA, single point based EEMD-SVD and single point based EMD-SVD method were used to denoise the siginal of sensor P1. The XGBoost classification models were trained and tested in the same way as described above, and the test results are shown in Tables 3 and 4.

**Table 3:** Classification and testing indicators of the effective information extracted by the single point based AVMD-SSA under different working conditions.

Operating mode	Single point based AVMD-SSA		
	Accuracy rate	recall	F1 metric
C1	0.9744	1.0000	0.9870
C6	0.8125	0.9630	0.8814
C11	1.0000	0.8750	0.9333
C16	0.8387	0.8387	0.8387
C21	0.8889	0.9697	0.9275
C26	0.9118	0.7949	0.8493
Precision		0.9050

**Table 4:** Classification and testing indicators of the effective information extracted by the single point based EEMD-SVD and the EMD-SVD under different working conditions.

Operating mode	Single point based EEMD-SVD			Single point based EMD-SVD		
	Accuracy rate	Recall	F1 metric	Accuracy rate	Recall	F1 metric
C1	0.9744	1.0000	0.9870	0.9744	1.0000	0.9870
C6	0.8065	0.9259	0.8621	0.7188	0.8519	0.7797
C11	1.0000	0.8750	0.9333	0.9032	0.8750	0.8889
C16	0.7222	0.8387	0.7661	0.7222	0.8387	0.7761
C21	0.8788	0.8788	0.8788	0.8611	0.9394	0.8986
C26	0.8788	0.7436	0.8056	0.8846	0.5897	0.7077
Precision		0.8750			0.8450	

As shown in Table 2, the precision of test classification results using the noise reduction proposed in this paper is 0.9200. As shown in Tables 3 and 4, the precision of test classification results using the the traditional single point based AVMD-SSA, single point based EEMD-SVD and single point based EMD-SVD noise reduction are 0.9050, 0.8750, and 0.8450, respectively. Hence, the method based on tensor tool proposed in this paper can greatly improve the differentiation degree of the signal under different damage conditions and can improve the accuracy of subsequent structure damage diagnosis.

## Conclusion

The conventional noise reduction method based on single measurement point vibration response data has low accuracy and efficiency, and it is difficult to adaptively distinguish the effective information and the useless noise component of the tensor. This paper has researched the research on the synchronous noise reduction method of vibration response data of multiple measurement points of hydraulic structures. The specific research content and results are as follows:

1. The Crank–Nicholson-Like criterion is introduced, and the core tensor and three factor matrix updating method of low-rank tensor are studied. The preliminary noise reduction method of spatiotemporal tensor data based on factor matrix filter set is proposed.
2. The time weight modified Dynamic Time Warping theory and the curvature smoothing algorithm combination model with a balance factor is constructed to realize the effective information mining of vibration response.

## Data Availability

The datasets used and/or analyzed during the current study available from the corresponding author on reasonable request.
